# Type 2 diabetes patients’ preferences and willingness to pay for lifestyle programs: a discrete choice experiment

**DOI:** 10.1186/1471-2458-13-1099

**Published:** 2013-11-29

**Authors:** Jorien Veldwijk, Mattijs S Lambooij, Paul F van Gils, Jeroen N Struijs, Henriëtte A Smit, G Ardine de Wit

**Affiliations:** 1 National Institute for Public Health and the Environment (RIVM), Center for Nutrition, Prevention and Health Services, PO Box 1 (101), Bilthoven, BA 3720, The Netherlands; 2 University Medical Center Utrecht, Julius Center for Health Sciences and Primary Care, Utrecht, The Netherlands

**Keywords:** Discrete choice experiment, Preferences, Diabetes mellitus type 2, Lifestyle program, Participation rate, Willingness to participate, Willingness to pay

## Abstract

**Background:**

Participation rates of lifestyle programs among type 2 diabetes mellitus (T2DM) patients are less than optimal around the globe. Whereas research shows notable delays in the development of the disease among lifestyle program participants. Very little is known about the relative importance of barriers for participation as well as the willingness of T2DM patients to pay for participation in such programs. The aim of this study was to identify the preferences of T2DM patients with regard to lifestyle programs and to calculate participants’ willingness to pay (WTP) as well as to estimate the potential participation rates of lifestyle programs.

**Methods:**

A Discrete Choice Experiment (DCE) questionnaire assessing five different lifestyle program attributes was distributed among 1250 Dutch adults aged 35–65 years with T2DM, 391 questionnaires (31%) were returned and included in the analysis. The relative importance of the program attributes (i.e., meal plan, physical activity (PA) schedule, consultation structure, expected program outcome and out-of-pocket costs) was determined using panel-mixed logit models. Based on the retrieved attribute estimates, patients’ WTP and potential participation rates were determined.

**Results:**

The out-of-pocket costs (β = −0.75, P < .001), consultation structure (β = −0.46, P < .001) and expected outcome (β = 0.72, P < .001) were the most important factors for respondents when deciding whether to participate in a lifestyle program. Respondents were willing to pay €128 per year for individual instead of group consultation and €97 per year for 10 kilograms anticipated weight loss. Potential participation rates for different lifestyle-program scenarios ranged between 48.5% and 62.4%.

**Conclusions:**

When deciding whether to participate in a lifestyle program, T2DM patients are mostly driven by low levels of out-of-pocket costs. Thereafter, they prefer individual consultation and high levels of anticipated outcomes with respect to weight loss.

## Background

Participation rates of lifestyle programs among type 2 diabetes mellitus (T2DM) patients are less than optimal around the globe [1,2]. Yet, there appears to be notable delays in the development of the disease and the onset of diabetes-related complications among participants in different lifestyle programs [1]–[5]. Driven by these results, combined with the increasing prevalence of T2DM and high disease-specific mortality rates [6,7], lifestyle interventions have been included in standardized care protocols for the treatment of T2DM patients in several countries [8]–[10]. However, in general, the participation rates of such lifestyle programs among T2DM patients are unsatisfactory, ranging from 20-70% of those eligible for such programs [1,2,11]. Suboptimal participation rates are especially worrisome among the growing population of T2DM patients aged 35–65 years. Lifestyle changes among patients within this specific age group are expected to have a greater and more long-term impact on disease progress than among older T2DM patients [12]. Moreover, these relatively young T2DM patients suffer less from serious diabetes-related physical or medical restrictions [13,14] or from other chronic diseases or disabilities [15,16]. Therefore, younger T2DM patients are relatively suitable candidates for participation in a lifestyle program.

In order to obtain a better insight into the motives of T2DM patients for participating in lifestyle interventions, various studies were conducted that resulted in an extensive list of barriers for participation in lifestyle interventions as reported by T2DM patients [17]–[21]. However, very little is known about the relative importance of such factors for these patients, while it seems reasonable to assume that not all of the factors are of equal importance in the decision-making process regarding the participation in a lifestyle program. Previous research has shown that this also applied with regard to lifestyle programs in other target populations [22]–[25].

A second omission in many studies on the willingness to participate in lifestyle programs is that the costs of lifestyle programs are often not included. If lifestyle interventions were to be implemented in ‘real life’, participants in such programs would have to pay at least part of the program costs out-of-pocket. Whereas participation in programs within a research setting tends to be free of charge. Taking into consideration that these programs need to be (partly) financed by the participants, it is worthwhile to examine the amount of money that potential participants would be willing to pay. Previous research among a non-diabetic population showed that the 582 individuals at a high risk of developing T2DM were willing to pay out-of-pocket for a lifestyle intervention [23]. These rates varied between $63 and $5 per month for a three-year course depending on the diet restrictions, hours of exercise, hour of counseling, use of medication, the goal that was set with respect to weight loss and the percentage expected reduction in T2DM risk [23].

Once we have obtained a better insight into and knowledge of program-related factors that are crucial for the participation of T2DM patients in these lifestyle programs as well as for their willingness to pay (WTP), recommendations can be made as to what type of program would most likely be preferred by its potential users. These recommendations can be taken into account when developing new lifestyle programs, thus increasing their reach, and hence their public health benefit.

The aim of this study was to identify the preferences of diabetes type 2 patients for different characteristics of lifestyle programs. Based on these preferences, participants’ willingness to pay (WTP) as well as the potential participation rates of different lifestyle programs have been assessed.

## Methods

### Participants and recruitment

Within the Netherlands, generic diabetes care is arranged in care groups. A care group is a legal entity formed by multiple health care providers (however, these are often exclusively general practitioners (GPs)) [26]. A random selection of care groups (per province of the Netherlands) was contacted to distribute the questionnaire among T2DM patients aged 35–65 years who were not suffering from any serious diabetes-related complications (i.e., cardiovascular diseases, nephropathy, retinopathy, neuropathy) and who were registered with that care group. In total five care groups, located within five different provinces of the Netherlands, participated. These care groups distributed a total number of 1250 questionnaires to all the eligible patients within their care groups, 391 (31.3%) of which were completed and included in the analysis. Due to confidentiality agreements with the care groups that distributed the questionnaires, no reminder letters could be sent. As a result, there was no non-response information available to empirically test whether responders differed from non-responders with respect to their demographic and disease-specific characteristics. The Dutch National Ethics Board (Central Committee on Research involving Human Subjects) concluded that formal testing by a medical ethical committee was not necessary, as T2DM patients were only required to complete an anonymous questionnaire once, which is in accordance with the guidelines laid down in the Declaration of Helsinki.

The mean age of the final study population was 57.2 years, more than half of the respondents were male (57.4%), almost all respondents were Dutch nationals (94.1%), and about half of the population (49.6%) completed higher secondary education or lower general professional education (Coded ‘medium’ in Table 1). On average, the participants were diagnosed with T2DM 6.1 years ago and they had a mean HbA_1c_ of 49.9 mmol/mol. Of all the respondents, 37.8% was overweight (indicated by a BMI between 25 and 30 kg/m^2^) and 41.1% was obese (indicated by a BMI higher than 30 kg/m^2^). The majority of the respondents reported no complications (78.3%) and no other chronic conditions (99.7%). 19% of the respondents reported that they did not use any form of medication, 67.2% reported that they used glucose lowering medication in the form of pills, 4.6% reported that they injected insulin, and 9.2% reported that they used both. Almost all respondents (94.1%) reported that their primary health care contact was their GP and not a specialist in secondary care (e.g., an internist). Self-management measures were applied by approximately half of this population; 45.8% reported that they monitored their HbA_1c_ at home and 50.3% reported that they keep a T2DM diary.

**Table 1:** General characteristics and psychosocial determinants of the study population (n = 391)

		**Mean (SD)**	**Percentage**
Age (n = 385)		57.2 (6.4)	
Gender (n = 390)	Male		57.4
Educational level (n = 379)	Low		31.1
	Medium		49.6
	High		19.2
Ethnicity (n = 390)	Dutch		94.1
Duration of diabetes (years) (n = 382)		6.1 (5.5)	
HbA_1c_ (mmol/mol) (n = 101)		49.9 (16.6)	
Primary health care contact (n = 187)	GP		94.1
Medication (n = 390)	None		19.0
	Oral glucose lowering medication		67.2
	Insulin		4.6
	Both		9.2
Complications present (n = 303)			21.7
Chronic condition present (n = 390)			0.3
Weight category* (n = 364)	Underweight		0.2
	Normal weight		19.5
	Overweight		37.8
	Obese		41.1
EQ5d score (n = 391)		0.91 (0.19)	
Self-management (n = 386)	monitoring HbA_1c_ at home		45.8
	Keeping a T2DM diary		50.3
What is your opinion concerning lifestyle programs in general? (n = 388)	Very useful or useful		47.2
	Not useful at all		2.6
Do you think you are able to complete a lifestyle program that endures 1 year, without dropping out? (n = 391)	Certainly or probably		33.5
	Certainly not		18.2
Would your partner, friends and/or family support you if you would participate in a lifestyle program? (n = 391)	Certainly or probably		66.7
	Certainly not		4.9
Would you like to participate in a lifestyle program? (n = 391)	Certainly or probably		22.6
	Certainly not		19.4

*Respondents were categorized as underweight if their BMI < 20 kg/m^2^, normal weight if BMI 20–25 kg/m^2^, overweight if BMI 25-30 kg/m^2^ and obese if BMI >30 kg/m^2^.

In total 47.2% of this population reported that they considered lifestyle programs to be useful and 66.7% thought that their partner, family or friends would support their participation in a lifestyle program, 33.5% felt that they would be able to complete such a lifestyle program and 22.6% would actually like to participate in such a program (Table 1).

### Discrete choice experiment

DCEs are becoming a frequently used tool in public health research to estimate the average participation rates of interventions or medical treatments and to provide knowledge about the components of the programs that determine the participation rates [27,28]. The DCE methodology is based on the Random Utility Theory and assumes that any intervention or treatment can be described by its characteristics (i.e., attributes; such as frequency of consultation). The individual’s preference for an intervention or treatment is dependent on the levels (e.g., weekly or monthly consultation) of those attributes [27,28]. By varying the levels of the attributes, different scenarios are constructed. Respondents are provided with at least two scenarios (i.e., choice tasks) simultaneously, they then have to choose the scenario that they prefer most. Each respondent is asked to complete a series of such choice tasks. In the end, conclusions can be drawn regarding the components that constitute an intervention that is most preferred by its potential users.

### Attributes, levels and design

The attributes and levels included in the current study were determined in a stepwise manner, which subsequently included a literature review, expert interviews and focus group interviews with T2DM patients. First, a list of barriers for participating in a lifestyle intervention by T2DM patients was compiled based on previously published literature [17]–[21]. Second, the list of barriers thus obtained was discussed during expert interviews with a physician, a dietician and a scientist with a specific interest in diabetes care. These expert interviews were conducted in order to [1] shorten the list of potential attributes and [2] to ensure that the attributes and levels were consistent with current practice. As a third step, four focus group interviews were conducted with 24 T2DM patients in order to ensure that [1] the most important attributes for the decision-making process of T2DM patients were included in the DCE and [2] proper levels were appointed to each of the attributes. Focus group interviews were conducted according to Krueger and colleagues [29]. This process led to the inclusion of five attributes (meal plan, physical activity (PA) schedule, consultation structure, expected outcomes and out-of-pocket costs) with three levels (Table 2).

**Table 2:** The attributes and levels that were included in this discrete choice experiment

**Attributes**	**Level 1**	**Level 2**	**Level 3**
**Meal plan: a plan, which describes the aims of the participants with respect to improvements in their diet, developed by the participants of the program together with a coach**			
	Flexible: primarily based on the participants’ own initiatives and ideas	General: includes general information on a healthy diet and provides example recipes	Elaborate: a patient tailored schedule that is completely prepared by the lifestyle coach
**Physical activity (PA) schedule: a plan, which describes the aims of the participants with respect to improvements in their PA behavior, developed by the participants of the program together with a coach**			
	Flexible: primarily based on the participants’ own initiatives and ideas	General: includes general information on PA, and provides example exercises	Elaborate: a patient tailored schedule that is completely prepared by the lifestyle coach
**Consultation structure: the composition of the consults with the coach**			
	Individually	Groups with 5 other T2DM patients	Groups with 10 other T2DM patients
**Expected outcomes: the results, in terms of weight loss and physical fitness expected by the respondent after completion of a lifestyle program**			
	No weight loss but feeling more healthy	5 kilograms of weight lost and feeling more healthy	10 kilograms of weight lost and feeling more healthy
**Out-of-pocket costs: patients may have to pay (part) of the program costs out-of-pocket**			
	75 euro per year	150 euro per year	225 euro per year

Based on the selected attributes and levels, NGene 1.0 (ChoiceMetrics, 2011) software was used to develop a D-efficient design with 18 unique choice tasks [30,31]. To limit the burden of respondents, NGene divided these 18 choice tasks over two sets of nine choice tasks, each set of nine choice tasks was disseminated among half of the study population. Besides choosing one of the lifestyle programs presented, participants could also choose an opt-out solution. This opt-out option was included because, in real life, people can also choose not to participate in a lifestyle program. Table 3 presents an example of a choice task as included in the questionnaire of this study. Before completing these choice tasks, respondents were provided with an extensive explanation of the meaning of all attributes and levels as well as an explanation about how to deal with a choice task, accompanied by an example. Every choice task starts with the question: ‘Imagine that your GP or nurse practitioner advises that you participate in a lifestyle program for a period of one year. In which situation would you prefer to participate, situation 1 or situation 2? If you do not wish to participate in either of the situations, you can tick the box “none”. A questionnaire containing additional questions was added to the DCE, for further details on this questionnaire see Additional file 1.

**Table 3:** Example of a choice task

	**Situation 1**	**Situation 2**	**None**
**Meal plan**	Flexible	General	None
**Physical activity schedule**	General	Elaborate	None
**Consultation structure**	Individual	In groups of 5 patients	None
**Expected outcome**	5 kg weight loss and feeling more healthy	10 kg weight loss and feeling more healthy	None
**Out-of-pocket costs**	150 euro per year	150 euro per year	0 euro per year
Tick the box of the situation that you prefer:	0	0	0

The draft questionnaire was pilot tested among a subgroup (n = 20) of the study population to ensure that the wording used in the questionnaire was correct and understood by the target population. Most of the pilot tests were performed by means of a postal questionnaire, respondents were requested to mark every question or answering category that they did not understand or found hard to grasp and they were asked to provide suggestions for improvement. Moreover, three think aloud pilot tests were conducted to obtain more insight into how the respondents approached answering the choice tasks. No changes in the attributes and/or levels were deemed necessary based on the results of this pilot study. Moreover, based on the pilot-test data, sample size calculations were performed to ensure that significant differences for each attribute could be detected at a 5% level [30,32].

### Statistical analyses

NLogit 4.0 (Econometric Software, 2007) was used to construct the panel-mixed-logit (Panel-MIXL) models that were estimated within this study. When using such models, results are adjusted for the panel structure (i.e., multilevel structure) of the data. As every respondent completed nine choice tasks, their answers may be correlated, which is then accounted for. Equation 1 was tested using these models.

(1) $$\begin{array}{l} U=V+\varepsilon =\beta_{0}+\beta_{1}*\text{flexible meal plan}+\beta_{2}*\text{elaborate meal plan}\\ +\beta_{3}\text{flexible PA schedule}\;+\beta_{4}*\text{elaborate PA schedule}\\ +\beta_{5}*\text{consultation in groups of}\;5+\beta_{6}*\text{consultation in groups of}10\\ +\beta_{7}*\text{expected outcome}+\beta_{8}*\text{out of pocket costs}+\varepsilon \end{array}$$

V describes the measurable utility of a specific lifestyle program based on the attributes that were included in the DCE. β_0_ represents the alternative specific constant and β_1_ – β_8_ are the attribute estimates that indicate the relative importance of each attribute. Based on the model fit (AIC and Chi-square tests), the constant and the expected outcome attributes were set as random parameters (both with a normal distribution).

All nonlinear parameters were recorded into effect codes. In contrast to dummy coding, this coding procedure codes the reference category −1, so the sum of the effect coded attributes is always 0. The coefficient for the reference category is therefore −1*(β_effect code 1_ + β_effect code 2_) [33,34].

In order to calculate patients’ marginal willingness to pay (WTP), the negative of the out-of-pocket attribute was used as a measure of the marginal utility of money. The ratio of either attribute estimate to this negative of the out-of-pocket attribute provides an estimation of patients’ WTP concerning that specific attribute [28,35]. Moreover, the potential participation rates (choice probabilities) of a program that consists of a specific set of attributes was estimated. Since both the constant and the expected outcome attribute were included as random parameters in the analyses, choice probabilities could not be calculated directly, therefore a simulation was used [28,33]. The mean participation rates of all simulations (n = 1000) were estimated by taking the average of all simulated participation rate probabilities, which were calculated as 1/(1 + exp^-v^).

For a more detailed description of the statistical methods used in this paper see Additional file 2.

## Results

### Patient preferences

Most of the attribute estimates were significant, indicating that they were important for T2DM patients when choosing whether to participate in a lifestyle program (Table 4). Participants did not have any distinct preferences with regard to the meal plan. However, they did prefer a general PA schedule above a flexible PA schedule. Participants reported a preference for individual consultation, as compared to consultation in groups of 10 patients. The greater the expected outcome in terms of weight loss, the more willing participants were to participate and higher out-of-pocket costs led to a decrease in their willingness to participate.

**Table 4:** T2DM patients’ preferences for a lifestyle program: the attribute estimates of the Panel-MIXL model

**Attribute**		**Beta value**	**SE**
Constant	Mean	0.11	0.13
	Standard deviation	2.61*	0.71
Meal plan	Flexible	0.11	0.07
	General (ref)	−0.04	0.06
	Elaborate	−0.06	0.06
PA schedule	Flexible	−0.13*	0.06
	General (ref)	0.02	0.01
	Elaborate	0.11	0.06
Consultation structure	Individual (ref)	0.50	0.08
	Groups of 5	−0.04	0.06
	Groups of 10	−0.46***	0.08
Expected outcome (10 kg)	Mean	0.72***	0.16
	Standard deviation	1.53*	0.51
Out-of-pocket costs (€100)		−0.75***	0.08

*significant at p < .05; ***significant at p < .001.

Since the magnitude of the beta values depends highly on the coding of the attributes, attributes were recorded into the same coding scale (all attribute levels were coded between −1 and 1) to enable the assessment of their relative importance. The results of the recoded analysis are not shown because they show a high degree of overlap with the results presented in Table 4 and provide betas that are difficult to interpret especially concerning the outcome and costs attributes. The results reveal that, based on the value of its coefficient, the out-of-pocket costs were the most decisive factor for respondents in determining whether they wanted to participate in a lifestyle program. This attribute was followed by the consultation structure and expected outcome. The least important factor in the decision-making process was the operationalization of the PA schedule.

The significant coefficient of the standard deviation of the expected outcome attribute indicates that there is indeed a high preference heterogeneity among respondents concerning the amount of weight loss they anticipate before starting the intervention.

Finally, the opt-out option was chosen in 46.3% of the choice tasks and 23.5% of the respondents chose to opt-out in every choice task. Patient preferences did not change when these latter responders were excluded from the analysis.

### Willingness to pay

The WTP was calculated for the significant attributes only. The results show that respondents were willing to pay €21.0 (95% CI: €11.3; €30.7) for a switch from a flexible to a general PA schedule. Respondents were willing to pay an extra €127.8 (95% CI: €106.0; €149.7) per year for a lifestyle program organized via individual consultation instead of consultation with a group of 10 other patients. Respondents were willing to pay €96.8 (95% CI: €85.2; €108.4) per year for 10 kg anticipated weight loss.

### Potential participation rate

A lifestyle program that consists of all of the least preferred attribute levels (flexible meal plan, flexible PA schedule, consultation in groups of 10, no weight loss) was set as the ‘base’ model. This program showed the lowest participation rate (48.5%) (Table 5). The potential participation rate increased if this ‘base’ model was adapted to the identified patient preferences (change to 51.5%-57.4%). The most preferred program, which includes a flexible meal plan, general PA schedule, individual consultation and a 10 kg weight loss, resulted in an estimated potential participation rate of 62.4%.

**Table 5:** Expected participation rates for different lifestyle programs based on the attribute estimates of the Panel-MIXL model

	**Participation rates (%)**	**Explanation**
Base model	48.5	A program with a general meal plan, a flexible PA schedule, consultation in groups of 10 and no weight loss
PA schedule	51.5	Base model with a general PA schedule
Consultation structure	54.8	Base model with individual consultation
Expected outcome	57.4	Base model with a 10 kg weight loss
Preferred program	62.4	A program with a flexible meal plan, a general PA schedule, individual consultation and a 10 kg weight loss

## Discussion

Our research is the first to demonstrate the relative importance of the factors that affect T2DM patients’ preferences for a lifestyle intervention program. Results showed that the out-of-pocket costs were the most crucial factor for T2DM patients when deciding whether to participate in a lifestyle program (i.e., patients preferred lowest costs). Moreover, a lifestyle program with a general physical activity component, individual consultation and large expected outcomes in terms of weight loss was preferred. T2DM patients were willing to pay €21, €128 and €97 per year respectively for a lifestyle program with these desired levels of the attributes (i.e., a general PA schedule, individual consultation, and 10 kg anticipated weight loss). Additionally, it was estimated that approximately 62% of the T2DM patients aged 35–65 years would participate in a lifestyle program with these preferred levels.

Though there is limited evidence regarding T2DM patients’ preferences for a lifestyle program, the current findings are comparable to those of previously conducted DCEs among other target populations describing different lifestyle interventions. The study by Johnson [23] found that individuals at a high risk of developing T2DM preferred a lifestyle program that specified anticipated weight loss over a program that did not describe any anticipated weight loss. They also found that programs with restrictive diets were disliked, that some sort of physical activity component was preferred, and that the respondents were willing to pay up to approximately $63 per month for a total of 36 months for participation in the lifestyle program they preferred most [23]. Roux and colleagues [25], as well as Owen and colleagues [24], reported that participants in a lifestyle program preferred personally oriented programs that included both a diet and some sort of PA component. The diet component in the program should not be too restrictive [24], and participants were willing to pay for participation [25].

Patients in the current study reported to be willing to pay up to €97 per year for every 10 kilograms of anticipated weight loss. Though this seems a promising argument to boost participation without significantly increasing costs, one could argue that, despite the linearity of the initial costs and expected outcome attributes, beyond a certain point, participants are no longer willing to pay an additional €97 for an increase of 10 kilograms of anticipated weight loss. This was also shown by Johnson and colleagues who demonstrated nonlinearity of the WTP [36].

Finally, results showed that a potential participation rate of 62% can be expected when attributes are operationalized in accordance with patient preferences. Current programs can be improved by organizing individual consultation and communicating clearly about the anticipated outcomes of the program (in terms of weight loss and degree of physical fitness of the participant). The participation rates found in this study show that T2DM patients are willing to participate if programs meet certain criteria. However, the participation rates do not exceed the most optimistic participation rates currently observed in lifestyle programs [1,2,11]. This suggests that T2DM patients have additional motives for not participating in a lifestyle program other than the tested characteristics of a lifestyle program. These motives may differ considerably between individuals; previous research already suggested tailored lifestyle programs to enhance patient commitment [37,38]. Tailoring such programs to individual patients can be costly and less feasible compared to generic programs. It should be explored which factors, other than consultation structure and clear communication with respect to expected outcomes, can be maintained over the total target population in order to limit the variation between programs and to keep costs as low as possible while at the same time increasing patient commitment.

Our conclusions are restricted by a number of limitations. The usable response rate was 31.3%. As the current study was questionnaire-based and participation was on a voluntary basis, selective non-response seems plausible. For instance, the number of non-Dutch patients was relatively low. This may be due to language difficulties, as a good command of the Dutch language is needed to complete the questionnaire and especially the DCE tasks. Therefore, generalizability with regard to preferences of non-Dutch patient groups remains limited. Besides, it could be the case that patients who already perceive their lifestyle as being healthy chose not to participate in this study. However, in real life, it is not likely that these patients would participate in a lifestyle program and therefore these patients are of limited interest for this specific study.

The current study included T2DM patients in the age group of 35–65 years, who do not suffer from severe diabetes-related complications. There is limited information on the representativeness of the current study population compared to the target population. Additional analyses of T2DM patients aged 35–65 in a large Dutch cohort study (EPIC-NL [39]) showed the same mean BMI values, but other characteristics could not be compared because of the specific inclusion and exclusion criteria of the current study (in particular, the exclusion of patients with severe diabetes-related complications in this study). Future research should be conducted among T2DM patients aged > 65 years without any restrictions with respect to the presence of diabetes-related complications to obtain more insight into the preferences of this subpopulation. Researchers should then take into account that the attributes of the current DCE might not be applicable within this new target population. Conducting such research would contribute to the insights into (the differences and similarities in) preferences of the entire T2DM patient population with respect to lifestyle programs.

## Conclusions

In conclusion, when deciding to participate in a lifestyle program, T2DM patients in the age group of 35–65 years are mostly driven by the out-of-pocket costs of a lifestyle program, the structure of the consultation and the expected outcome of the program. We therefore advise that lifestyle programs directed at T2DM patients should be set up based on individual consultation, while communicating about the expected outcomes of the program (in terms of weight loss) and keeping out-of-pocket costs as limited as possible.

## Competing interests

This research was funded by the Strategic Research fund of the National Institute of Public Health and the Environment. Sponsors were not part of the research group and therefore were not involved in conducting the research, writing the manuscript or submitting the final paper. None of the authors have stated that any competing interest exists.

## Authors’ contributions

JV prepared the questionnaire, conducted the expert interview and focus groups, designed the DCE, performed the data analysis and wrote the manuscript. MSL contributed to all phases mentioned above. PFG and HAS were involved in writing the research protocol and have both critically reviewed the manuscript. JNS assisted in the recruitment of the care groups that distributed the questionnaire and critically reviewed the manuscript. GAW was the project leader of this research project and was therefore involved in all stages of this study. All authors read and approved the final manuscript.

## Pre-publication history

The pre-publication history for this paper can be accessed here:

http://www.biomedcentral.com/1471-2458/13/1099/prepub

## Supplementary Material

Additional file 1 **Detailed description of the additional questionnaire.** Here a detailed description is provided about the content of the questionnaire that was distributed alongside the DCE questionnaire. Click here for file

Additional file 2 **Detailed description of the statistical methods.** Here a detailed description is provided about statistical background end methods that were used for the analysis in this study. Click here for file
