# Supplementary material for: Type 2 diabetes patients’ preferences and willingness to pay for lifestyle programs: a discrete choice experiment
Source: BMC Public Health. 2013 Nov 29;13:1099. doi: 10.1186/1471-2458-13-1099 (PMC3909291; doi:10.1186/1471-2458-13-1099)
Supplement: Additional file 1 — Detailed description of the additional questionnaire. Here a detailed description is provided about the content of the questionnaire that was distributed alongside the DCE questionnaire. [file 1471-2458-13-1099-S1.pdf]

Additional file 1: Detailed description of the additional questionnaire

<u>Questionnaire</u>

The questionnaire consisted of two parts. The first section of the questionnaire comprised 28 questions starting with questions concerning the participant's demographics, such as gender, age and educational level. Educational levels were categorized into low (primary school or lower general secondary education), medium (higher secondary education or lower general professional education) and high (i.e., bachelor or master's degree) educational level. Thereafter questions pertained to the patient's disease status: type of diabetes, disease duration since diagnosis, primary provider of care, use of medication, latest measured HbA_{1c} level, body height and weight, presence of complications or other chronic illnesses and self-management activities (i.e., self-measurement of HbA_{1c} levels and keeping a diabetes diary). This section of the questionnaire ends with the EuroQol-5D health status questionnaire (EQ-5D) (1) and the patient's attitude, social norm, self-efficacy and intention with respect to lifestyle programs in general (answers were given on a 5-point Likert scale). The second part of the questionnaire consists of the actual DCE as explained above.

References

1. Lamers LM, Stalmeier PF, McDonnell J, Krabbe PF, van Busschbach JJ. **[Measuring the quality of life in economic evaluations: the Dutch EQ-5D tariff]**. *Ned Tijdschr Geneeskd* 2005; 149: 1574-8.
