# Supplementary material for: Type 2 diabetes patients’ preferences and willingness to pay for lifestyle programs: a discrete choice experiment
Source: BMC Public Health. 2013 Nov 29;13:1099. doi: 10.1186/1471-2458-13-1099 (PMC3909291; doi:10.1186/1471-2458-13-1099)
Supplement: Additional file 2 — Detailed description of the statistical methods. Here a detailed description is provided about statistical background end methods that were used for the analysis in this study. [file 1471-2458-13-1099-S2.pdf]

Additional file 2: Detailed description of the statistical methods

Statistical analyses

NLogit 4.0 (Econometric Software, 2007) was used to construct the panel-mixed-logit (Panel-MIXL) models that were estimated within this study. When using such models, results are adjusted for the panel structure (i.e., multilevel structure) of the data. As every respondent completed nine choice tasks, their answers may be correlated, which is then accounted for. Equation 1.1 was tested using these models.

$V = \beta_0 + \beta_1$ * flexible meal plan + β_2 * elaborate meal plan + β_3 flexible PA schedule + β_4 * elaborate PA schedule + β_5 * consultation in groups of 5 + β_6 * consultation in groups of 10 + β_7 * expected outcome + β_8 * out-of-pocket costs [Eq. 1.1]

V describes the utility of a specific lifestyle program based on the attributes that were included in the DCE. β_0 represents the alternative specific constant and $\beta_1 – \beta_8$ are the attribute estimates that indicate the relative importance of each attribute. Interactions between the attribute estimates and demographic and background characteristics were examined to determine whether there were specific subgroups that reported different preferences. As no such subgroups could be identified, the analyses were not adjusted for demographic or other background characteristics.

All attributes were tested for linearity, all parameters that were found to be non-linear were recoded into effect codes. In contrast to dummy coding, this coding procedure codes the reference category -1, so the sum of the effect coded attributes is always 0. The coefficient for the reference category is therefore $-1*(\beta_{\text{effect code 1}} + \beta_{\text{effect code 2}})$. This makes it possible to compare the estimates of all attributes despite their categorization into effect codes (1, 2) as

opposed to dummy coding, which normally allows for comparison between the dummies only.

Based on the model fit (AIC and Chi-square tests), the constant of the model was set as a random parameter and the expected outcome attribute (both with a normal distribution) was also set as a random parameter. The expected outcome of the program may possibly show high preference heterogeneity among the participants also because of large differences in BMI between the respondents. The presence of preference heterogeneity does not necessarily indicate subgroups within a population (e.g., T2DM preference heterogeneity for the outcome attribute, does not automatically mean that patients with a high versus a low BMI choose substantially different preferences for all attributes).

In order to calculate the patient's marginal willingness to pay (WTP), the negative of the out-of-pocket attribute was used as a measure of the marginal utility of money. The ratio of either attribute estimate to this negative of the out-of-pocket attribute provided an estimation of the patient's WTP concerning that specific attribute (3, 4). Moreover, the potential participation rates (choice probabilities) of a program that consist of a specific set of attributes were estimated. Out-of-pocket costs were not included in these analyses, as the influence of out-of-pocket payments was already covered by calculating the WTP ratios. As both the constant and the expected outcome attributes were included as random parameters in the analyses, choice probabilities could not be calculated directly. A simulation was used to estimate the choice probabilities, given a certain set of attributes (1, 4). The mean participation rates of all simulations (n=1000) were estimated by taking the average of all simulated participation rate probabilities, which were calculated as $1/(1+\exp^{-V})$.

References

1. Louviere JJ, Hensher DA, Swait JD: *Stated Choice Methods; analysis and apllication*. Cambridge: Cambridge University Press; 2000.
2. Bech M, Gyrd-Hansen D. **Effects coding in discrete choice experiments**. *Health Econ* 2005; 14: 1079-83.
3. Revelt D, Train KE. **Mixed Logit with Repeated Choices: Households' Choices of Appliance Efficiency Level.** *The Review of Economics and Statistics* 1998; 80: 647-57.
4. Ryan M, Gerard K, Amaya-Amaya M: *Using Discrete Choice Experiments to Value Health and Health Care*. Dordrecht: Springer; 2008.
